# A new method for ranking *q*-rung ortho-pair fuzzy numbers and application

**DOI:** 10.1371/journal.pone.0327395

**Published:** 2025-07-11

**Authors:** Mengchuan Zhao, Yi Xiang, Yan Yang, K. E. Deng

**Affiliations:** 1 School of Mathematics, Sichuan University, Chengdu, Sichuan, China; 2 School of Mathematics and Statistics, Sichuan University of Science & Engineering, Zigong, Sichuan, China; 3 School of Sciences, Southwest Petroleum University, Chengdu, Sichuan, China; Amity University Haryana, INDIA

## Abstract

The effectiveness of the *q*-rung ortho-pair fuzzy multi-attribute decision-making method is primarily influenced by the *q*-rung ortho-pair fuzzy number ranking method. This paper conducts an in-depth analysis of the shortcomings of eight existing *q*-rung ortho-pair fuzzy number ranking methods. A refined approach to ranking q-rung ortho-pair fuzzy numbers is proposed, wherein the method synthesizes the effects of the q-power transformation applied to both membership and non-membership degrees, alongside an exponential adjustment component. This formulation ensures greater discrimination power and robustness in uncertain environments. This method addresses the issues of poor robustness and the inability to achieve a complete ranking in existing approaches. Finally, the proposed ranking approach is incorporated into a *q*-rung orthopair fuzzy multi-attribute decision-making framework and is subsequently employed to address a practical case involving the selection of an optimal warehouse location for an e-commerce enterprise.

## 1. Introduction

Prof. Zadeh [1] of the University of California pioneered fuzzy set theory in 1965, expanding the traditional concept of set membership by allowing membership values to range between 0 and 1. This approach introduced affiliation levels to describe the relationship between elements and the set. Within classical fuzzy set theory, the non-membership degree is typically derived as the complement of the membership degree, that is, by subtracting it from unity. This construction inherently couples the two values, thereby restricting their independent representation.

In response to this structural limitation, Atanassov (1986), a Bulgarian mathematician [2], introduced the framework of intuitionistic fuzzy sets (IFS), wherein the membership and non-membership degrees are treated as distinct quantities bounded within the unit interval [0, 1]. The residual uncertainty—termed the hesitation degree—is then formally defined as the difference between 1 and the sum of these two parameters. Intuitionistic fuzzy sets have since been widely applied across various disciplines, including engineering, computer science, mathematics, decision sciences, energy studies, environmental science, and the social sciences [3–9], due to their effectiveness in handling uncertain data.

Despite the continued development and refinement of intuitionistic fuzzy set theory, it remains inherently limited to cases where the sum of membership and non-membership degrees does not exceed one. This constraint precludes its application in more expressive uncertainty modeling. To address this issue, Yager (2013), a prominent American scholar, proposed the notion of *Pythagorean fuzzy sets* (PyFS) [10], wherein the squared sum of the membership and non-membership degrees is restricted to lie within the unit interval, thereby allowing greater representational flexibility. This refinement ensured that the sum of the squared membership and non-membership degrees remained within the unit interval [0,1]. Pythagorean fuzzy sets (PyFS) enhance the descriptive power available to decision-makers by accommodating situations that fall beyond the expressive limits of intuitionistic fuzzy sets. Nevertheless, their structure remains bounded by the constraint that the squared sum of membership and non-membership degrees must not exceed unity. As a result, PyFS may still fall short in modeling certain forms of uncertainty where this bound is naturally violated.

**Fig 1:**
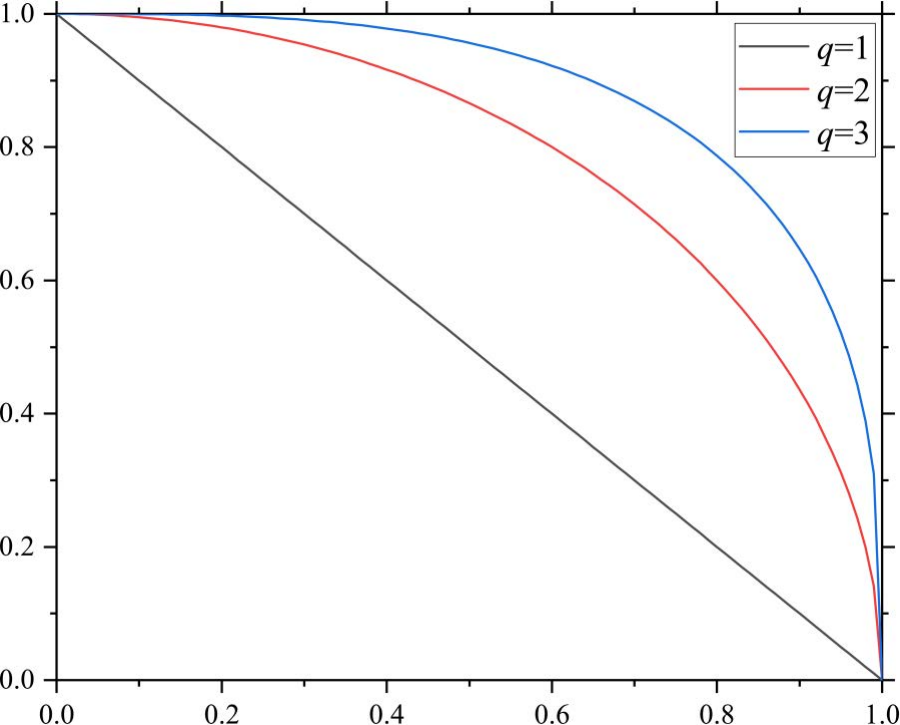
Comparison of the *q*-ROFS space for *q *= 1, 2, 3.

In 2016, Yager [11] further extended fuzzy set theory by introducing *q*-rung ortho-pair fuzzy sets (q-ROFSs), which ensure that the sum of the *q*-th power of membership and non-membership degrees remains within the unit interval. The *q*-ROFS framework generalizes IFS, PyFS and FFS: when *q* = 1, it reduces to an intuitionistic fuzzy set, and when *q* = 2, it corresponds to a Pythagorean fuzzy set. Furthermore, when *q* = 3, it transforms into a Fermatean fuzzy set. As *q* increases, the allowable decision space expands, providing greater flexibility for decision-making (Fig 1). The distinguishing feature of *q*-ROFSs lies in their ability to accommodate cases where the sum of membership and non-membership degrees exceeds 1 while still maintaining the constraint that their *q*-th power sum remains within the unit interval. This characteristic enhances decision-makers’ flexibility while mitigating the risk of information loss.

Although IFS and PyFS have proven effective across multiple domains, the emergence of *q*-ROFSs has further broadened their applications in engineering, computer science, energy studies, material science, environmental science, chemical engineering, and decision sciences ([12–30], [31,32]). The effectiveness of *q*-ROFS implementation largely depends on the ranking of *q*-rung ortho-pair fuzzy numbers (*q*-ROFNs), which has become a focal point in academic research.

Beginning in 2018, considerable scholarly attention has been devoted to the advancement of ranking methodologies for *q*-rung orthopair fuzzy numbers (*q*-ROFNs). In this context, Liu et al. (2018) introduced a suite of aggregation operators tailored to the *q*-ROFN framework [33], alongside a comparative mechanism for evaluating such fuzzy quantities. Peng et al. [34] introduced an innovative scoring mechanism to address Liu and Wang’s limitations in *q*-ROFN comparison. Du et al. [35] introduced a class of Minkowski-type distance measures tailored to *q*-rung orthopair fuzzy membership structures, thereby laying a mathematical foundation for the comparative analysis and ranking of fuzzy information pairs. Wei et al. [36] introduced *q*-rung ortho-pair fuzzy Heronian mean operators and proposed a novel score function to establish order relationships among *q*-ROFNs.

**Fig 2:**
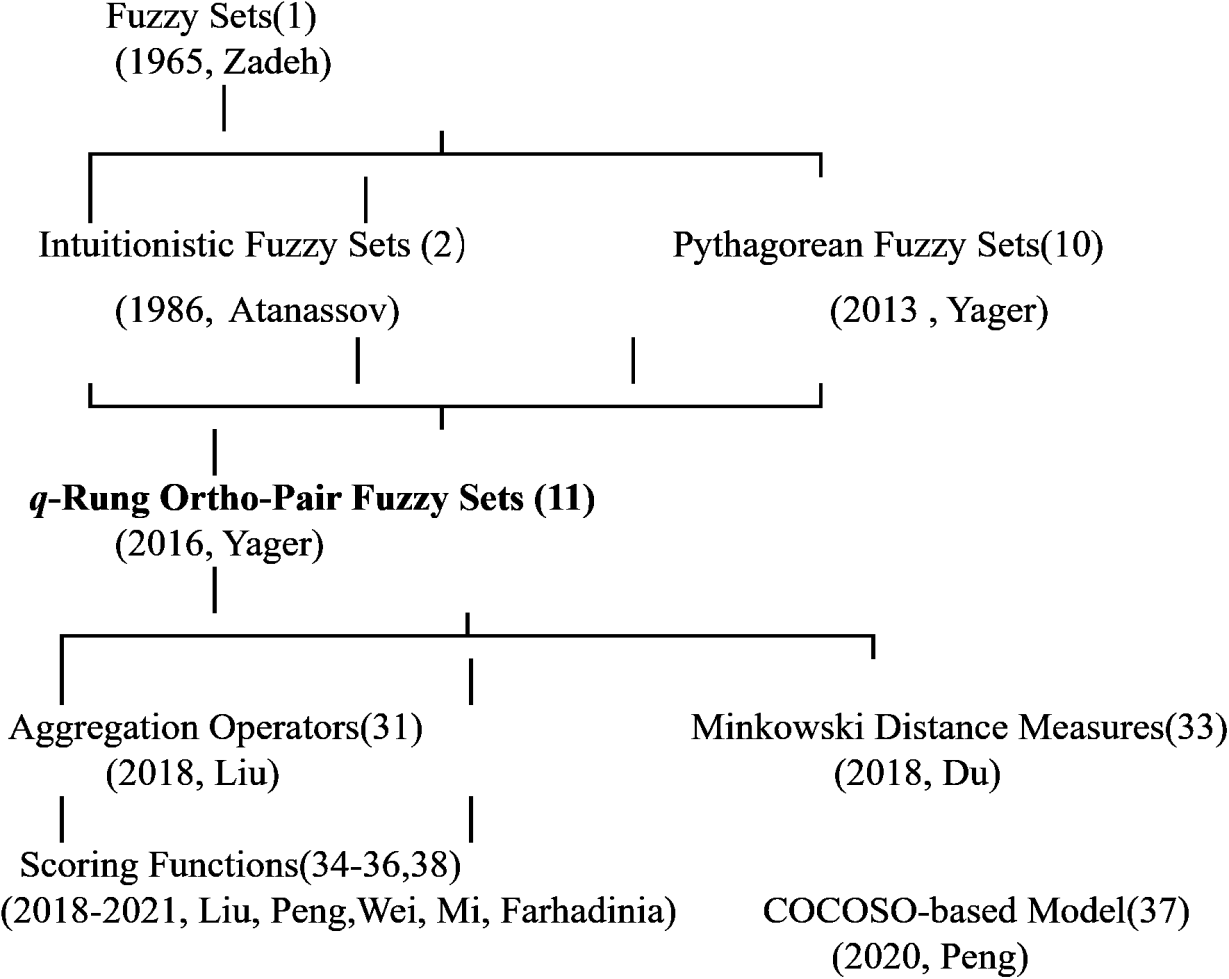
Development of *q*-Rung Ortho-Pair Fuzzy Numbers (*q*-ROFNs).

Further refinements emerged in 2019. Mi et al. [37] proposed a new *q*-ROFN scoring mechanism to overcome the limitations of Liu and Peng’s methods. That same year, Peng et al. [38] introduced another innovative scoring approach to resolve ranking inconsistencies in Liu and Peng’s techniques. In 2020, Peng et al.[39] developed a COCOSO-based fuzzy decision-making model to evaluate financial risks, incorporating a novel *q*-rung ortho-pair fuzzy scoring function to improve ranking accuracy. In 2021, Farhadinia et al. [40] introduced a parameterized scoring system for *q*-ROFSs, integrating a weighted mean approach to balance membership and non-membership degrees. The evolutionary trajectory of *q*-rung orthopair fuzzy numbers is illustrated in Fig 2.

Despite these advancements, many existing methods fail to fully capture the complexity of *q*-ROFNs, particularly in high-uncertainty environments. Common limitations include insensitivity to hesitation degrees, susceptibility to minor variations, and an inability to produce complete rankings. Addressing these shortcomings is essential, as effective *q*-ROFN ranking methods are crucial for improving decision-making across various domains. Table 1 categorizes our study in relation to existing work, clearly identifying the research gap addressed by our manuscript.

**Table 1:** Evolution of *q*-ROFN Scoring Mechanisms and Decision Models (Grouped by Limitations).

No.	Literature (s)	Method/ Model	Contribution	Main Limitations
1	Peng et al. [34], Peng et al. [38] Farhadinia et al. [40]	Improved scoring mechanisms	Addressed inconsistencies in earlier ranking methods	Incomplete or inconsistent ranking results
2	Du et al. [31], Wei et al. [32]	Distance-based/ mean operators for scoring	Developed ranking tools using Minkowski distance and Heronian mean	Low generalizability, strong reliance on expert-defined parameters
3	Mi et al. [33], Peng et al. [35],	Hybrid decision-making models (COCOSO, parameterized scores)	Integrated scoring with real-world decision-making frameworks	Sensitive to slight variations, lacks robustness
4	This paper	Natural exponent e Model	Introduced a unified and robust scoring framework	

This study aims to develop a novel ranking method for *q*-ROFNs that overcomes the limitations of existing approaches. The key objectives are as follows:

- To critically analyze eight existing *q*-ROFN ranking techniques and identify their limitations.
- To propose a novel ranking formula that integrates the *q*-rung power of the membership degree, the *q*-rung power of the non-membership degree, and an exponential factor.
- To validate the proposed ranking method by analyzing its four key properties and comparing its performance with existing techniques.
- To apply the proposed ranking method to real-world decision-making scenarios, particularly in e-commerce warehouse location selection.

The scope of this research encompasses theoretical advancements in *q*-ROFN ranking methods, their practical applications in decision sciences, and their impact on multi-criteria decision-making frameworks.

The structural organization of this paper proceeds according to the following logical sequence:

**Section 2,** we present a succinct exposition of the essential definitions, underlying principles, and notational framework associated with *q*-rung orthopair fuzzy sets. **Section 3** examines eight existing *q*-ROFN ranking techniques and highlights their limitations. **Section 4** introduces an advanced ranking framework for *q*-rung orthopair fuzzy numbers, wherein the design incorporates *q*-power transformations of both membership and non-membership degrees, augmented by an exponential adjustment term to enhance the model’s discriminative precision. Furthermore, four fundamental properties of the proposed method are formally examined. **Section 5** demonstrates the practical applicability of the proposed ranking approach through a case study on e-commerce warehouse location selection. Finally, **Section 6** summarizes the key findings and proposes directions for future research.

## 2. Preliminaries

In this section, we explore the fundamentals of IFS, PyFS, FFS and *q*-ROFS, as well as their interconnections and related definitions.

Let X be a nonempty universe of discourse. The following definitions outline several important generalizations of classical fuzzy sets.

**Definition 2.1** [2] An intuitionistic fuzzy set I on X is defined as:


$$I=\left\{\left\langle x,\mu_{I}\left(x\right),\nu_{I}\left(x\right)\right\rangle |x\in X\right\}$$

Where $\mu_{I}\left(x\right),\nu_{I}\left(x\right)\in \left[0,1\right]$ respectively denote the degrees of membership and non-membership of the element x, subject to the condition:


$$\mu_{I}\left(x\right)+\nu_{I}\left(x\right)\leq 1$$

The hesitation degree is defined as:


$$\pi_{I}\left(x\right)=1-\left(\mu_{I}\left(x\right)+\nu_{I}\left(x\right)\right)$$

The set of all intuitionistic fuzzy sets in the universe X is denoted as IFS(X). The ordered pair $\left(\mu_{I},\nu_{I}\right)$ is referred to as an intuitionistic fuzzy number (IFN).

**Definition 2.2** [10] A Pythagorean fuzzy set P on X is given by:


$$P=\left\{\left\langle x,\mu_{P}\left(x\right),\nu_{P}\left(x\right)\right\rangle |x\in X\right\}$$

Where $\mu_{P}\left(x\right),\nu_{P}\left(x\right)\in \left[0,1\right]$ and satisfy:


$$\mu_{P}^{2}\left(x\right)+\nu_{P}^{2}\left(x\right)\leq 1$$

The hesitation degree is:


$$\pi_{P}\left(x\right)=\sqrt{1-\left(\mu_{P}^{2}\left(x\right)+\nu_{P}^{2}\left(x\right)\right)}$$

The collection of all Pythagorean fuzzy sets in the universe X is represented as PFS(X).The ordered pair $\left(\mu_{P},\nu_{P}\right)$ is termed the Pythagorean fuzzy number (PFN).

**Definition 2.3 [11]** A Fermatean fuzzy set F on X is represented by:


$$F=\left\{\left\langle x,\mu_{F}\left(x\right),\nu_{F}\left(x\right)\right\rangle |x\in X\right\}$$

Where $\mu_{F}\left(x\right),\nu_{F}\left(x\right)\in \left[0,1\right]$ and the constraint:


$$\mu_{F}^{3}\left(x\right)+\nu_{F}^{3}\left(x\right)\leq 1$$

The corresponding hesitation degree is:


$$\pi_{F}\left(x\right)=\sqrt[{3}]{1-\left(\mu_{F}^{3}\left(x\right)+\nu_{F}^{3}\left(x\right)\right)}$$

The set of all Fermatean fuzzy sets in the universe X is denoted as FFS(X). The ordered pair $\left(\mu_{F},\nu_{F}\right)$ is known as the Fermatean fuzzy number (FFN).

**Definition 2.4 [11]** For a positive integer $q\in Z^{+}$, a *q*-rung ortho-pair fuzzy set Q on X is defined as:


Q={⟨x,μ(x),ν(x)⟩|x∈X}

where μ(x),ν(x)∈[0,1] and must satisfy:


$$\mu^{q}\left(x\right)+\nu^{q}\left(x\right)\leq 1,$$

The hesitation degree is computed as:


$$\pi \left(x\right)=\sqrt[{q}]{1-\left(\mu^{q}\left(x\right)+\nu^{q}\left(x\right)\right)},$$

which quantifies the uncertainty in assigning x to Q.

The collection of all *q-*rung orthopair fuzzy sets in the universe X is denoted as q−ROFS(X). The ordered pair (μ,ν) is referred to as the *q*-rung ortho-pair fuzzy number (*q*-ROFN).

**Remarks:**

(1) If q=1, the *q*-rung ortho-pair fuzzy set reduces to an intuitionistic fuzzy set.
(2) If q=2,the *q*-rung ortho-pair fuzzy set transforms into a Pythagorean fuzzy set.
(3) If q=3,the *q*-rung ortho-pair fuzzy set corresponds to a Fermatean fuzzy set.

**Definition 2.5** Let β=(μ,ν) be a *q*-ROFN. The maximum and minimum *q*-ROFNs are defined as follows:


$$\beta_{\max}=\left(1,0\right)$$

$$\beta_{\min}=\left(0,1\right)$$

**Definition 2.6** Let $\beta_{1}=\left(\mu_{1},\nu_{1}\right)$ and $\beta_{2}=\left(\mu_{2},\nu_{2}\right)$ denote two *q*-rung orthopair fuzzy numbers (*q*-ROFNs). The Minkowski distance between $\beta_{1}$ and $\beta_{2}$ is defined as follows:


$$d_{M}\left(\beta_{1},\beta_{2}\right)={\left(\frac{1}{2}{\left|\mu_{1}-\mu_{2}\right|}^{p}+\frac{1}{2}{\left|\nu_{1}-\nu_{2}\right|}^{p}\right)}^{1/p},p\geq 1$$

By assigning distinct values to the parameter p, the Minkowski distance reduces to well-known specific metrics: when p=1,it yields the **Hamming distance**; for p=2,it corresponds to the **Euclidean distance**; and in the limiting case as p→∞, it converges to the **Chebyshev distance**.

The principal abbreviations and notational symbols employed throughout this manuscript are systematically summarized in Tables 2 and 3, respectively.

**Table 2:** Abbreviations of core concepts utilized in the manuscript.

Concepts	Abbreviation
Intuitionistic fuzzy sets	IFS
Intuitionistic fuzzy number	IFN
Pythagorean fuzzy sets	PyFS
Pythagorean fuzzy number	PFN
Fermatean fuzzy sets	FFS
Fermatean fuzzy number	FFN
*q-*rung orthopair fuzzy sets	*q*-ROFS
*q*-rung ortho-pair fuzzy number	*q*-ROFN
*q*-rung ortho-pair fuzzy weighted Geometric	*q*-ROFWG
Multi-criteria decision-making	MCDM

**Table 3:** Mathematical symbols and notations employed in the manuscript.

Concepts	Symbols
Intuitionistic fuzzy set	*I*
Universe set	*X*
Pythagorean fuzzy set	*P*
Fermatean fuzzy set	*F*
*q*-rung ortho-pair fuzzy set	*Q*
Degree of membership	*μ*(*x*)
Degree of non-membership	*ν*(*x*)
Hesitation degree	*π*(*x*)
Minkowski distance	*d*_*M*_(*β*_1_, *β*_2_)
Score function	*S*_1_ ~ *S*_8_, *S*, *S*_1_, *S*_*P*_, *S*_*F*_
Accuracy function	*H*(*β*)
Set of alternatives	*L* = {*L*_1_, *L*_2_, *L*_3_, ..., *L*_*n*_}
*Set of attributes*	*A* = {*A*_1_, *A*_2_, *A*_3_, ..., *A*_*n*_}
*Weight vector*	*w*

## 3. Comparison of existing ranking methods

This section examines eight existing methods for ranking *q*-ROFNs and discusses their limitations.

**Definition 3.1 [33]** Let β=(μ,ν) be a *q*-ROFN. The score function $S_{1}$ of β is defined by: $S_{1}\left(\beta \right)=\mu^{q}-\nu^{q}$

where q is a positive integer and β∈ *q*-ROFN. The range of $S_{1}\left(\beta \right)$ lies within [−1,1]. Additionally, the corresponding accuracy function H(β) is given as:

$H\left(\beta \right)=\mu^{q}+\nu^{q}$, with H(β)∈[0,1].

**Algorithm 3.1** Considering these two principles, for any two *q*-ROFNs $\beta_{1}=\left(\mu_{1},\nu_{1}\right)$ and $\beta_{2}=\left(\mu_{2},\nu_{2}\right)$, ranking is determined as follows:

- $\beta_{1}$ is preferred over $\beta_{2}$ if $S_{1}\left(\beta_{1}\right)>S_{1}\left(\beta_{2}\right)$.
- $\beta_{1}$ is ranked lower than $\beta_{2}$ if $S_{1}\left(\beta_{1}\right)<S_{1}\left(\beta_{2}\right)$.

If $S_{1}\left(\beta_{1}\right)=S_{1}\left(\beta_{2}\right)$, then

- $\beta_{1}$ is preferred over $\beta_{2}$ if $H\left(\beta_{1}\right)>H\left(\beta_{2}\right)$.
- $\beta_{1}$ is ranked lower than $\beta_{2}$ if $H\left(\beta_{1}\right)<H\left(\beta_{2}\right)$.
- $\beta_{1}$ and $\beta_{2}$ are considered equivalent if $H\left(\beta_{1}\right)=H\left(\beta_{2}\right)$.

While **Algorithm 3.1** is effective in ranking values, it has notable limitations. The score function $S_{1}$ accurately orders uncertain options, yet Peng et al. [34] identified that both $S_{1}$ and the accuracy measure H fail to consider hesitation effects, leading to potential misclassification. To address this, **Definition 3.2** introduces an improved score function $S_{2}$, ensuring robustness against minor perturbations. However, excessive sensitivity to small numerical fluctuations (e.g., below 0.0001) remains a concern.

**Definition 3.2 [34–40]** Let β=(μ,ν) represent a *q*-ROFN. A series of refined score functions have been introduced. These score functions are formally defined in Table 4.

**Table 4:** Definitions of $S_{2}$ to $S_{8}$.

score function	Value range
$S_{2}\left(\beta \right)=\mu^{q}-\nu^{q}+\left(\frac{e^{\mu^{q}-\nu^{q}}}{e^{\mu^{q}-\nu^{q}}+1}-\frac{1}{2}\right)\pi^{q}$	$S_{2}\left(\beta \right)\in \left[-1,1\right]$
$S_{3}\left(\beta \right)=\frac{d_{M}\left(\beta ,\beta_{\min}\right)}{d_{M}\left(\beta ,\beta_{\min}\right)+d_{M}\left(\beta ,\beta_{\max}\right)}$	$S_{3}\left(\beta \right)\in \left[0,1\right]$
$S_{4}\left(\beta \right)=\frac{1}{2}\left(1+\mu^{q}-\nu^{q}\right)$	$S_{4}\left(\beta \right)\in \left[0,1\right]$
$S_{5}\left(\beta \right)=\frac{2+\mu^{q}-\nu^{q}}{\left(2-\mu^{q}+\nu^{q}\right)\times \left(1+\pi^{q}\right)}$$=\frac{2+\mu^{q}-\nu^{q}}{\left(2-\mu^{q}+\nu^{q}\right)\times \left(2-\mu^{q}-\nu^{q}\right)}$.	$S_{5}\left(\beta \right)\in \left[\frac{1}{3},3\right]$
$S_{6}\left(\beta \right)=\frac{\mu^{q}-2\nu^{q}-1}{3}+\frac{\lambda }{3}\left(\mu^{q}+\nu^{q}+2\right),\lambda \in \left[0,1\right]$	$S_{6}\left(\beta \right)\in \left[-1,1\right]$
$S_{7}\left(\beta \right)=\left(\mu^{q}-\nu^{q}\right)-\ln\left(1+\pi^{q}\right)$	$S_{7}\left(\beta \right)\in \left[-1,1\right]$
$S_{8}\left(\beta \right)=\mu^{q}+\lambda \left(1-\mu^{q}-\nu^{q}\right)$$=\mu^{q}+\lambda \pi^{q}$$=\left(1-\lambda \right)\mu^{q}+\lambda \left(1-\nu^{q}\right),\lambda \in \left(0,1\right)$.	$S_{8}\left(\beta \right)\in \left[0,1\right)$

**Algorithm 3.2** For two *q*-ROFNs $\beta_{1}=\left(\mu_{1},\nu_{1}\right)$ and $\beta_{2}=\left(\mu_{2},\nu_{2}\right)$:

- $\beta_{1}$ is preferred over $\beta_{2}$ if $S_{i}\left(\beta_{1}\right)>S_{i}\left(\beta_{2}\right)$;
- $\beta_{1}$ is ranked lower than $\beta_{2}$ if $S_{i}\left(\beta_{1}\right)<S_{i}\left(\beta_{2}\right)$;
- $\beta_{1}$ and $\beta_{2}$ are considered equivalent if $S_{i}\left(\beta_{1}\right)=S_{i}\left(\beta_{2}\right),$ for i=2,3,4,...,8.

**Fig 3:**
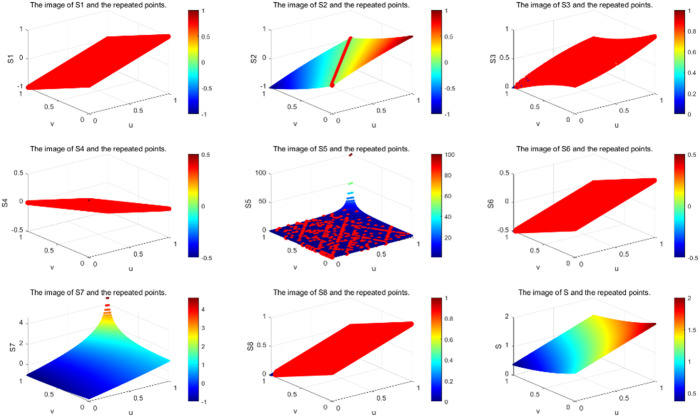
The score function plots of $S_{1}$ to $S_{8}$ and S for q=1.

**Fig 4:**
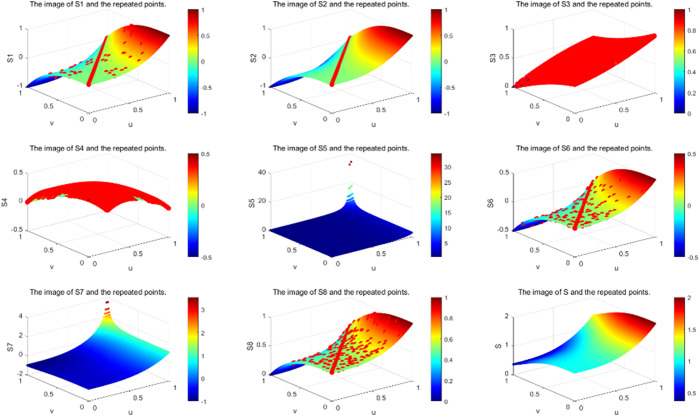
The score function plots of $S_{1}$ to $S_{8}$ and S for q=2.

**Fig 5:**
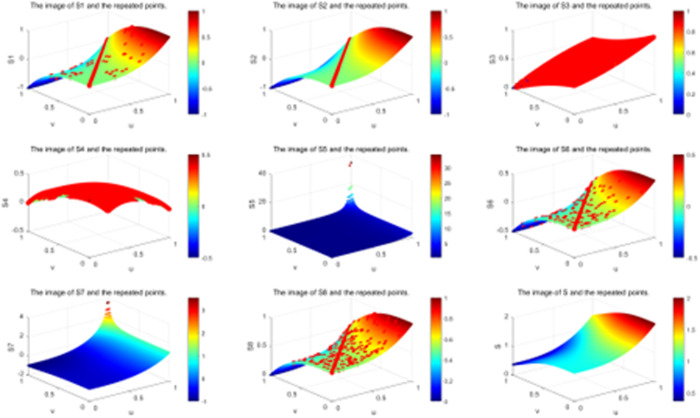
The score function plots of $S_{1}$ to $S_{8}$ and S for q=3.

Furthermore, it is evident that the score functions $S_{2}$ and $S_{4}$ encounter similar issues when μ=ν. Specifically, these functions fail to fully rank *q*-ROFNs. Due to the continuous presence of various membership and non-membership values, the score function remains unchanged. Likewise, $S_{5}$ and $S_{7}$ are also incapable of completely ranking *q*-ROFNs. As stated in **Definition 2.6**, the *q* parameter is not considered in the distance computation. When utilizing distance-based metrics for *q*-ROFNs., certain limitations arise. For instance, given that μ=ν, the score function $S_{3}\left(\beta \right)$ consistently equals 0.5, meaning that *q*-ROFNs are always assigned the same position when μ=ν. Consequently, the closeness index $S_{3}$ is unable to distinguish between different *q*-ROFNs. Due to the presence of multiple variations in membership and non-membership statistics, the score function remains unchanged. For example, when μ=ν (with λ=0.5,∀q), the score function $S_{6}$ remains at zero, while $S_{8}$ consistently maintains a value of 0.5. In summary, both $S_{6}$ and $S_{8}$ are incapable of fully ranking *q*-ROFNs. The score function results of $S_{1}$ to $S_{8}$ are presented in Figs 3–5.

In conclusion, the primary shortcomings of the existing techniques include their failure to account for hesitation levels and their excessive sensitivity to minor variations. Additionally, these methods are unable to achieve a complete ranking of *q*-ROFNs and suffer from identical scoring functions when μ=ν.

## 4. A novel approach for ranking *q*-rung orthopair fuzzy numbers

Considering the limitations of existing ranking techniques, this work introduces a new ranking criterion based on the following key components: [1] the *q*-th power of the membership grade $\mu^{q}$, [2] the *q*-th power of the non-membership grade $v^{q}$, and [3] the exponential function as a weighting modulator. The proposed method exhibits a concise mathematical formulation characterized by computational tractability. The introduction of this method effectively addresses the shortcomings of the eight existing approaches, such as the inability to achieve complete ranking and poor robustness. Furthermore, we investigate four key properties of this proposed score function.

**Definition 4.1** Given a *q*-ROFN β=(μ,ν), its associated score function S is defined by the following expression:


$$S\left(\beta \right)=\mu^{q}+e^{-v^{q}}$$ (4.1)

We refer to S(β) as the score value of *q*-ROFNs, satisfying


$$\frac{1}{e}\leq S\left(\beta \right)\leq 2$$

(1) When q=1,the score function S(β) reduces to $S_{I}\left(\beta \right)$ for IFNs:


$$S_{I}\left(\beta \right)=\mu +e^{-\nu }$$ (4.2)

(2) When q=2,the score function S(β) transforms into $S_{P}\left(\beta \right)$ for PFNs:


$$S_{P}\left(\beta \right)=\mu^{2}+e^{-\nu^{2}}$$ (4.3)

(3) When q=3,the score function S(β) corresponds to $S_{F}\left(\beta \right)$ for FFNs:


$$S_{F}\left(\beta \right)=\mu^{3}+e^{-\nu^{3}}$$ (4.4)

From equation (4.1), we can deduce the following properties:

**Property 4.1 (Monotonicity)** The score function S(β) monotonically increases with respect to μ and decreases with respect to ν.

**Proof:** The partial derivatives of S(β) with respect to μ and ν are computed as follows:


$$\frac{\partial S\left(\beta \right)}{\partial \mu }=\frac{\partial \left(\mu^{q}+e^{-\nu^{q}}\right)}{\partial \mu }=q\mu^{q-1}\geq 0$$

$$\frac{\partial S\left(\beta \right)}{\partial \nu }=\frac{\partial \left(\mu^{q}+e^{-v^{q}}\right)}{\partial \nu }=-qv^{q-1}e^{-v^{q}}\leq 0$$

Thus, S(β) increases as μ increases and decreases as ν increases.

This property indicates that an alternative possessing a higher degree of membership and a lower degree of non-membership will be prioritized in the ranking. In conclusion, the ranking procedure for *q*-ROFNs is outlined as follows:

**Algorithm 4.1**

For two *q*-ROFNs $\beta_{1}=\left(\mu_{1},\nu_{1}\right)$ and $\beta_{2}=\left(\mu_{2},\nu_{2}\right)$:

- $\beta_{1}$ is superior to $\beta_{2}$ if $S\left(\beta_{1}\right)>S\left(\beta_{2}\right)$.
- $\beta_{1}$ is inferior to $\beta_{2}$ if $S\left(\beta_{1}\right)<S\left(\beta_{2}\right)$.
- $\beta_{1}$ is equivalent to $\beta_{2}$ if $S\left(\beta_{1}\right)=S\left(\beta_{2}\right)$.

This algorithm can also be applied to ranking IFNs, PFNs, and FFNs by setting q=1,2,3. We are now capable of addressing the issues unresolved by the techniques discussed in Section 3.

**Property 4.2 (Boundedness)** For any *q*-ROFN β=(μ,ν), the sore function S(β) satisfies the following relations:

(1) $\frac{1}{e}\leq S\left(\beta \right)\leq 2$;
  (2) S(β)=2 if and only if $\beta =\beta_{\max}=\left(1,0\right)$;
(3) $S\left(\beta \right)=\frac{1}{e}$ if and only if $\beta =\beta_{\min}=\left(0,1\right)$.

**Proof:** According to **Property 4.1**, S(β) monotonically increases with respect to μ and decreases with respect to ν. Hence, the maximum value of S(β) is achieved when $\beta =\beta_{\max}=\left(1,0\right)$, yielding S(β)=2. Conversely, the minimum value is attained when $\beta =\beta_{\min}=\left(0,1\right)$, yielding $S\left(\beta \right)=\frac{1}{e}$.

Therefore, the range of S(β) is confirmed as


$$\frac{1}{e}\leq S\left(\beta \right)\leq 2$$

**Property 4.3 (Uniqueness)** Let $\beta_{1}=\left(\mu_{1},\nu_{1}\right)$ and $\beta_{2}=\left(\mu_{2},\nu_{2}\right)$ be two *q*-ROFNs. If $S\left(\beta_{1}\right)=S\left(\beta_{2}\right)$, then $\beta_{1}=\beta_{2}$.

**Proof:** According to **Definition 4.1**, the following equation holds:


$$S\left(\beta_{1}\right)=\mu_{1}^{q}+e^{-v_{1}^{-q}}=\mu_{2}^{q}+e^{-v_{2}^{-q}}=S\left(\beta_{2}\right)$$

Let $\Delta_{u}=\mu_{1}^{q}-\mu_{2}^{q}$ and $\Delta_{\nu }=e^{-v_{2}^{-q}}-e^{-v_{1}^{-q}}.$ Then:

(1) The function $\mu^{q}$ is strictly monotonic in its increase with respect to μ.

If $\mu_{1}>\mu_{2}$, then $\Delta_{u}>0$.

If $\mu_{1}<\mu_{2}$, then $\Delta_{u}<0$.

If $\mu_{1}=\mu_{2}$, then $\Delta_{u}=0$.

(2) The function $e^{-v^{q}}$ is strictly monotonically decreasing with respect to ν.

If $\nu_{1}>\nu_{2}$, then $\Delta_{\nu }>0$.

If $\nu_{1}<\nu_{2}$, then $\Delta_{\nu }<0$.

If $\nu_{1}=\nu_{2}$, then $\Delta_{\nu }=0$.

From these conditions, three cases arise:

Case 1: $\Delta_{u}=\Delta_{\nu }=0\Rightarrow \mu_{1}=\mu_{2},\nu_{1}=\nu_{2}\Rightarrow \beta_{1}=\beta_{2}.$

Case 2: $\Delta_{u}=\Delta_{\nu }>0$, meaning $\mu_{1}>\mu_{2}$ and $\nu_{1}>\nu_{2}$. Due to the monotonicity of $\mu^{q}$ and $e^{-v^{q}}$, $\Delta_{u}$ and $\Delta_{\nu }$ vary in opposite directions, making it impossible for them to be equal.

Case 3: $\Delta_{u}=\Delta_{\nu }<0$, implying $\mu_{1}<\mu_{2}$ and $\nu_{1}<\nu_{2}$. The same reasoning applies, leading to a

contradiction.

Since the only possible scenario satisfying the equality $S\left(\beta_{1}\right)=S\left(\beta_{2}\right)$ is when $\Delta_{u}=\Delta_{\nu }=0$, we conclude that $\beta_{1}=\beta_{2}$.

Thus, **Property 4.3** demonstrates that the proposed method ensures a fully ordered ranking of *q*-ROFNs, a crucial advantage over conventional ranking techniques.

**Property 4.4 (Yager’s criterion)** Let $\beta_{1}=\left(\mu_{1},\nu_{1}\right)$ and $\beta_{2}=\left(\mu_{2},\nu_{2}\right)$ be two *q*-ROFNs. The following conditions hold:

(1) If $\mu_{1}\geq \mu_{2}$ and $\nu_{1}<\nu_{2}$, then $S\left(\beta_{1}\right)>S\left(\beta_{2}\right)$.
(2) If $\mu_{1}<\mu_{2}$ and $\nu_{1}\geq \nu_{2}$, then $S\left(\beta_{1}\right)<S\left(\beta_{2}\right)$.

**Proof:** Based on Property 4.1, it can be clearly demonstrated that this property holds.

In Table 5, we summarize the properties satisfied by the eight existing ranking methods and the proposed method. It is evident that none of the existing methods satisfy the uniqueness property, which explains their inability to achieve a complete ranking. However, the method proposed in this study ensures uniqueness, enabling a comprehensive ordering of *q*-ROFNs. From Table 6 and Figs 3–5, it can be observed that the scoring functions $S_{1}-S_{4}$, $S_{6}$ and $S_{8}$ exhibit relatively strong robustness. However, they fail to achieve complete ordering, and their function values remain constant when μ=ν. In contrast, the scoring functions $S_{5}$ and $S_{7}$ demonstrate weaker robustness and are also incapable of achieving complete ordering. Notably, only the method proposed in this paper satisfies both strong robustness and the ability to achieve complete ordering while ensuring that the scoring function value is not invariant when μ=ν.

**Table 5:** Comparison of properties between existing and proposed approaches.

Methods	Monotonicity	Boundedness	Uniqueness	Yager’s criterion
$S_{1}$	✓	✓	✗	✓
$S_{2}$	✓	✓	✗	✓
$S_{3}$	✗	✓	✗	✗
$S_{4}$	✓	✓	✗	✓
$S_{5}$	✓	✓	✗	✓
$S_{6}$	✓	✓	✗	✓
$S_{7}$	✓	✓	✗	✓
$S_{8}$	✓	✓	✗	✓
Proposed method	✓	✓	✓	✓

**Note**: ✓ indicates adherence to the property, while ✗ denotes non-compliance.

**Table 6:** Scoring function robustness, complete ordering, and consistency when μ=ν.

Methods	Robustness	The Score Function Differs When μ=ν	Total Ordering
*S*_1_	✓	✗	✗
*S*_2_	✓	✗	✗
*S*_3_	✓	✗	✗
*S*_4_	✓	✗	✗
*S*_5_	✗	✓	✗
*S*_6_	✓	✗	✗
*S*_7_	✗	✓	✗
*S*_8_	✓	✗	✗
Proposed method	✓	✓	✓

**Note**: ✓ indicates the presence of the property, while ✗ denotes the absence of the property.

## 5. Applications of *q*-ROFNs in E-commerce warehouse location selection

Given that *q*-ROFNs extend the concepts of IFSs, PFSs, and FFSs, they exhibit superior capacity in addressing practical issues compared to existing models. To illustrate the effectiveness of *q*-ROFNs, this section explores their application in the selection of e-commerce warehouse locations.

**Algorithm 5.1** Consider a collection of alternatives $L=\left\{L_{1},L_{2},L_{3},...,L_{n}\right\}$ and a corresponding set of evaluations $A=\left\{A_{1},A_{2},A_{3},...,A_{n}\right\}$, where each $A_{i}$ is derived from predefined attributes. The central objective of this decision-making framework is to establish a total order among the alternatives, thereby identifying the optimal choice according to the specified criteria. The procedure is implemented through the following sequence of operations:

**Step 1:** Construct a decision matrix by assigning specific values to each option based on selected attributes, represented as *q*-ROFNs.

**Step 2:** Compile data from decision-makers into a decision matrix.

**Step 3:** Evaluate the collective data.

**Step 4:** Organize and analyze the collective data to determine the most suitable option.

**Example 5.1** A certain e-commerce platform company aims to select an optimal warehouse location. To facilitate this decision, the company considers the following four key factors:

(1) **Market Demand**

**Customer Distribution** The location should be close to the main customer base to shorten delivery times and enhance customer experience.

**Purchasing Power** Areas with high purchasing power should be selected to ensure high warehouse utilization.

(2) **Transportation Conditions**

**Accessibility** The warehouse should be situated near transportation hubs such as highways, railways, and ports to ensure efficient logistics.

**Delivery Efficiency** Transportation conditions directly affect delivery speed and costs, making them a key consideration in site selection.

(3) **Land and Construction Costs**

**Land Price** Selecting areas with lower land costs can reduce initial investment.

**Construction Costs** Consideration of warehouse construction and maintenance expenses is essential to ensure long-term operational feasibility.

(4) **Labor Resources**

**Labor Supply** Ensure that there is an adequate supply of labor to support warehouse operations.

**Labor Costs** Choose regions with lower labor costs to control operational expenses.

To facilitate decision-making, the e-commerce company invited experts to evaluate three potential warehouse locations based on the four aforementioned attributes. For each evaluation attribute, the experts furnished their judgments utilizing *q*-ROFNs as the representation format.

The attribute set is defined as

A={marketdemand,transportationconditions,treatmentresults,landandconstructioncosts,laborresources} The corresponding weight vector for these attributes is given by:


$$w={\left(0.3,0.3,0.2,0.2\right)}^{T}$$

Decision-makers then expressed their preferences using *q*-ROFNs, as outlined in **Table 7**.

**Table 7:** Expert evaluation matrix.

	$A_{1}$	$A_{2}$	$A_{3}$	$A_{4}$
$L_{1}$	(0.6,0.8)	(0.6,0.9)	(0.7,0.7)	(0.8,0.6)
$L_{2}$	(0.8,0.6)	(0.7,0.6)	(0.5,0.8)	(0.6,0.8)
$L_{3}$	(0.7,0.8)	(0.4,0.8)	(0.5,0.7)	(0.6,0.9)

**Step 1:** The presented data clearly demonstrates that every value listed in Table 7 is exclusively expressed as *q*-ROFNs for n=3. This finding highlights the limitations of IFSs and PFSs in handling such data, thereby underscoring the superiority of *q*-ROFNs over existing models.

**Step 2:** To consolidate the data in Table 7, the *q*-ROFWG operator [33] is applied, yielding the following compiled data:

$\beta_{1}=\left(0.6554,0.8055\right)$, $\beta_{2}=\left(0.6605,0.7057\right)$, $\beta_{3}=\left(0.5365,0.8141\right)$

**Step 3:** The scoring values for the aggregated data are computed using Equation (4.1) as follows:

$S\left(\beta_{1}\right)=0.6554^{3}+e^{-0.8055^{3}}=0.8745$,

$S\left(\beta_{2}\right)=0.6605^{3}+e^{-0.7057^{3}}=0.9918$,

$S\left(\beta_{3}\right)=0.6554^{3}+e^{-0.8055^{3}}=0.7374$.

**Step 4:** Since *S*(*β*_2_) > *S*(*β*_1_) > *S*(*β*_3_), it follows that candidate *L*_2_ is the most favorable among the three alternatives based on the evaluated attributes.

## 6. Conclusions

This study aimed to develop an enhanced ranking technique for *q*-ROFNs by addressing the limitations of eight existing ranking methods. A novel ranking approach was introduced, integrating affiliation degrees, non-affiliation degrees, and an exponential function to refine the ranking process of *q*-ROFNs. The effectiveness of the proposed method was rigorously validated through mathematical proofs, underscoring its superiority over conventional techniques.

### 6.1 Findings

The principal findings of this study can be summarized as follows:

(1) The proposed ranking method demonstrates superior stability and reliability compared to conventional ranking techniques, making it more robust for practical applications.
(2) Unlike existing ranking approaches, the newly developed method achieves total ranking, ensuring a clear differentiation among all *q*-ROFNs without ties.
(3) The method was successfully applied to e-commerce warehouse location selection, illustrating its practical utility in real-world decision-making scenarios.

### 6.2 Research limitations

Despite its advantages, the proposed ranking method has certain limitations:

(1) The method is specifically designed for *q*-ROFNs and may not be directly applicable to other fuzzy set models, which constrains its generalizability.
(2) Although the approach has been mathematically validated, its effectiveness across various application domains requires further empirical evaluation.
(3) The computational complexity of the ranking process necessitates additional analysis, particularly in large-scale decision-making problems.

### 6.3 Recommendations for Future Research

To enhance the adaptability and practical applicability inherent in the proposed ranking framework, future research may consider the following directions:

(1) The development of ranking mechanisms that allocate differentiated exponents to the membership and non-membership degrees. Such an approach would facilitate the natural extension of the method to more generalized fuzzy models, including [1,2]-fuzzy sets, (m,n)-fuzzy sets, and self-reciprocal fuzzy sets (SR-fuzzy sets) as referenced in [41–43].
(2) Investigating the incorporation of HyperFuzzy sets [44] into the ranking framework to enhance its adaptability in dealing with highly intricate or ambiguous decision-making environments.
(3) Extending the methodology to a broader range of application domains, including but not limited to engineering, computer science, energy systems, materials science, environmental studies, chemical engineering, and decision science [22–30,45,46].
(4) Improving the computational efficiency of the ranking algorithm, particularly with respect to its scalability and performance in large-scale multi-criteria decision-making (MCDM) problems.

Addressing these research directions will further refine the ranking methodology and expand its applicability to a broader range of uncertain decision-making scenarios.

## Supporting information

S1 Data Raw data supporting the numerical examples and comparative results discussed in the manuscript. (ZIP)
